# Food Contaminant Zearalenone and Its Metabolites Affect Cytokine Synthesis and Intestinal Epithelial Integrity of Porcine Cells

**DOI:** 10.3390/toxins7061979

**Published:** 2015-05-29

**Authors:** Daniela E. Marin, Monica Motiu, Ionelia Taranu

**Affiliations:** Laboratory of Animal Biology, National Institute for Research and Development for Biology and Animal Nutrition, Calea Bucuresti No. 1, Balotesti, Ilfov 077015, Romania; E-Mails: motiu.monica@ibna.ro (M.M.); ionelia.taranu@ibna.ro (I.T.)

**Keywords:** intestine, zearalenone, metabolites, swine, epithelial cells

## Abstract

The intestinal epithelium is the first barrier against food contaminants. Zearalenone (ZEN) is an estrogenic mycotoxin that was identified as a common contaminant of cereal grains and food and feedstuffs. In the present study, we have investigated the *in vitro* effects of ZEN and some of its metabolites (α-ZOL, β-ZOL) in concentrations of 10–100 µM on a swine epithelial cell line: Intestinal porcine epithelial cells (IPEC-1). We demonstrated that both ZEN metabolites were more toxic for IPEC cells as resulted from the XTT test, while for doses lower than 10 µM, only β-ZOL showed a more pronounced cytotoxicity *versus* epithelial cells as resulted from neutral red assay. ZEN has no effect on TER values, while α-ZOL significantly decreased the TER values, starting with day 4 of treatment. β-ZOL had a dual effect, firstly it induced a significant increase of TER, and then, starting on day 6, it induced a dramatic decrease of TER values as compared with on day 0. Concerning the cytokine synthesis, our results showed that ZEN has a tendency to increase the synthesis of IL-8 and IL-10. By contrast, α- and β-ZOL decreased the expression of both IL-8 and IL-10, in a dose dependent manner. In conclusion, our results showed that ZEN and its metabolites differently affected porcine intestinal cell viability, transepithelial resistance and cytokine synthesis with important implication for gut health.

## 1. Introduction

The intestinal epithelium is the first barrier against food contaminants [1,2]. Intestinal epithelial cells could be exposed to various concentrations of toxic substances like mycotoxins after the ingestion of contaminated food or feed [3,4]. Zearalenone (ZEN) is an estrogenic mycotoxin that can be produced by several *Fusarium* species and it was identified as a common contaminant of cereal grains and food and feedstuffs [5]. Beside its effect on reproductive tract, ZEN is known to alter the intestinal villous structure [6,7] and to reduce the expression of junction proteins [8].

Many studies have shown that ZEN is metabolized in different animal tissues, the major metabolites being alpha-zearalenol (α-ZOL) and beta-zearalenol (β-ZOL) [9,10]. For instance, in the duodenum and jejunum of sows, zearalenone was reduced in the presence of Nicotinamide adenine dinucleotide phosphate (NADPH) to both α- and β-ZOL [11]. Even the amount of α-ZOL formed by the gastrointestinal tissue was lower than that produced by the liver, the contribution of all gastrointestinal tissue to the production of α-ZOL was estimated to be comparable to that of the liver due to the large mass of gastrointestinal tissues [10].

The reproductive system is the major target of ZEN toxicity [12], but ZEN has been shown to also be immunotoxic, hepatonephrotoxic and an enhancer of lipid peroxidation [13].

Depending on the tissular target, ZEN and ZEN metabolites showed different toxicity. For example, immunocellular toxicity was not correlated with the estrogenic potency of ZEN and its derivatives, α-ZOL being the most toxic [13,14]. However, to the best of our knowledge, there are few studies investigating the effects of ZEN and its metabolites on the gastrointestinal tract [15].

Through the diet rich in cereals, a pig is exposed to the intoxication with the mycotoxins [16]. Also, pigs are very sensitive to the mycotoxin exposure [17]. From the agricultural point of view, the pig is an important animal farm species, and the economic losses could be important for farmers if their animals are confronted with feed contaminated with mycotoxins [18]. There is a need for data concerning the toxicity of ZEN in farm animals, and European Food Safety Authority (EFSA) recommends an increase of investigations *in vivo* and *in vitro*, especially at the cellular and molecular levels, in order to establish a comprehensive limit of tolerance in feed.

In the present study, we investigated the *in vitro* effects of ZEN and some of its metabolites (α-ZOL and β-ZOL) on several specific key parameters of the epithelium, such as cell viability, cytokines’ synthesis and epithelium integrity, crucial for the intestine barrier function of the epithelium. The focus was on renewal, permeability and immune response in a swine epithelial cell line derived from the small intestine of a newborn un-suckled piglet: IPEC-1.

The intestine represents the first barrier against the ingested food/feed contaminants. The disruption of the intestinal barrier increases penetration of feed contaminants and pathogens, with repercussions for the entire organism. In order to maintain an effective barrier function, epithelia need to exist in a constant state of regeneration. The epithelial surface is established by at least two mechanisms: epithelial proliferation and epithelial maturation/differentiation [19]. Epithelial cells can produce cytokines and chemokines, which are crucial for the recruitment of the immune cells, and several cytokines are constitutively expressed by intestinal cells playing a role in the influx of immune cells into the mucosa, in cell growth and homeostasis [20]. It was shown that several mycotoxins affect the cytokine production in epithelium, but there are few data regarding the effect of ZEN on intestinal cytokine production. Epithelial integrity is crucial in maintaining the intestinal barrier, its decrease may lead to intestinal inflammation [8].

## 2. Results and Discussion

### 2.1. Effect of ZEN and Its Metabolites on Cell Viability

**Figure 1:**
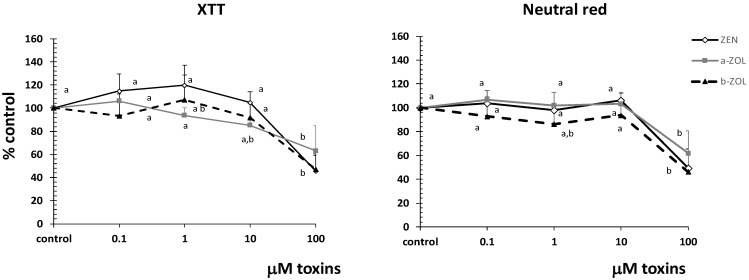
Effect of zearalenone and its metabolites on the IPEC-1 cells viability. The ability of IPEC cells to proliferate was measured using XTT and NR assays. Data are expressed as mean ± SEM from four independent experiments. Two way ANOVA tests were performed to determine the effect of toxin treatment. ^a,b^ indicate significant differences (*p* < 0.05) between control and different concentrations of ZEN and its metabolites.

In order to maintain an effective barrier function, epithelia need to exist in a constant state of regeneration [15]. The intestinal epithelial cells are very active cells, subjected to a continuous renewal, and the cell viability under the exposure of different toxins represents an important marker of the toxicity of different food and feed contaminants. For this purpose, the viability of the IPEC-1 cells under ZEN or ZEN metabolites action was evaluated using 2,3-Bis-(2-Methoxy-4-Nitro-5-Sulfophenyl)-2H-Tetrazolium-5-Carboxanilide (XTT) and neutral red (NR). As shown in Figure 1, the highest dose (100 µM) decreased significantly the cell viability by 54.3% (ZEN), 36.9% (α-ZOL), and 52.8% (β-ZOL) in the case of XTT test and by 50.4% (ZEN), 38.0% (α-ZOL), and 53.9% (β-ZOL) for the NR test.

Indeed, our previous results [13,14] showed that concentrations of ZEN metabolites required to reduce by 50% the cell viability of porcine blood lymphocytes and neutrophils were lower than those required for ZEN. Also, in RAW264.7 macrophages, the decrease of cell viability produced by β-ZOL was higher than that of α-ZOL, and it induced cell death mainly by apoptosis rather than necrosis [21]. It seems that apoptosis is the major cause of ZEN and ZEN metabolites-induced cell death [21,22]. Additionally, exposure of human bronchial epithelial cells to ZEN resulted in impaired response to DNA damage and cell cycle arrest [23]. Apparently, the mechanisms by which ZEN and its metabolites mediate their apoptotic cytotoxic effects appear to be different according to the cell type and the exposed toxins [21]. For example, in the mouse Leydig cells, the activation of an endoplasmic reticulum stress pathway plays a key role in ZEN-induced apoptosis [24], and reactive oxygen species (ROS) are the main upstream signal leading to increased ZEN mediated neurotoxicity in SH-SY5Y neuroblastoma cells [25]. On the other side, matrix metalloproteinases loss and nuclear translocation of apoptosis-inducing factor are the critical downstream events for ZEN metabolites-mediated apoptosis in macrophages, while the activation of p53, JNK or p38 kinase by ZEN metabolites is the main upstream signal required for the mitochondrial alteration of Bcl-2/Bax signaling pathways and intracellular ROS generation [21].

### 2.2. Effect of ZEN and Its Metabolites on Transepithelial Electrical Resistance

The gastrointestinal epithelium function as a barrier involved in the protection of the organism against the penetration of different contaminants and bacterial pathogens from the intestinal lumen into the systemic circulation [26].

**Figure 2:**
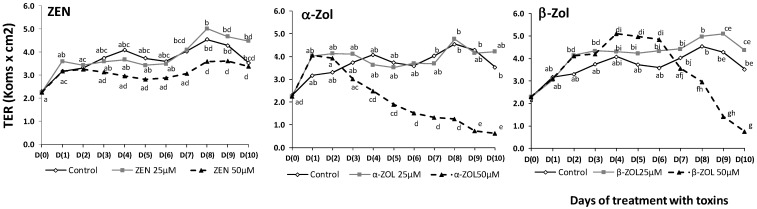
Effect of ZEN and its metabolites on transepithelial electrical resistance (TER) in IPEC-1 cells*.* Cells were grown and differentiated on inserts. At day 0, 25 and 50 µM ZEN and metabolites were added in the apical compartment, and TER was measured. TER values are expressed in kΩ × cm^2^ as mean ± SEM of four independent experiments. Two way ANOVA tests were performed to determine the effect of toxin treatment. ^a–g^ indicate significant differences (*p* < 0.05) between control and different concentrations of toxins.

TER can be considered a good indicator of the epithelial integrity and of the organization of the tight junction within epithelial cell monolayer [1]. The decrease of intestinal barrier integrity may lead to intestinal inflammation [8]. For this reason, we have assessed the effects of ZEN and its metabolites on TER of polarized porcine epithelial cell over a nine day period. The results are presented in Figure 2 and show different effects of mycotoxins on TER values depending on the toxin type and toxin concentration.

The low dose (25 µM) has no effect on TER as compared with the control, during the ten days of administration of the toxins. When a high dose of toxins (50 µM) was added to the IPEC-1 cells, different time effects were observed. ZEN has no effect on TER values while α-ZOL significantly decreased the TER, starting with day 4 of treatment. β-ZOL had a dual effect, it induced in the first time a significant increase of TER from 32% in the day 2 to 57% in the day 6, as compared with day 0. Starting with day 7, the TER values decreased (15%), and after 10 days of treatment, 50 µM of β-ZOL dramatically decreased the TER with 75% compared with day 0 (Figure 2). This TER decrease might result in an increase of intestinal permeability. Indeed, using a Millicell system, Pfeiffer *et al*. [27] has shown that 20 µM ZEN and α-ZOL were able to affect the apparent permeability coefficients of Caco-2 cells leading to their quick absorption from the intestinal lumen into the portal blood. The mechanisms responsible for the TER decrease seem to be related to the alteration of epithelium integrity through the tight junction proteins. In pregnant Sprague-Dawley rats, ZEN affected the villous structure and reduced the expression of junction proteins claudin-4, occludin and connexin43 (Cx43) in a dose-dependent manner [8]. However, other mechanisms such as the alteration in transcellular ions transport [28] may be involved in the TER observed variations and could be responsible for example for the early transiently increase of TER induced by β-ZOL.

### 2.3. Effect of ZEN and Its Metabolites on Cytokine Synthesis

**Figure 3:**
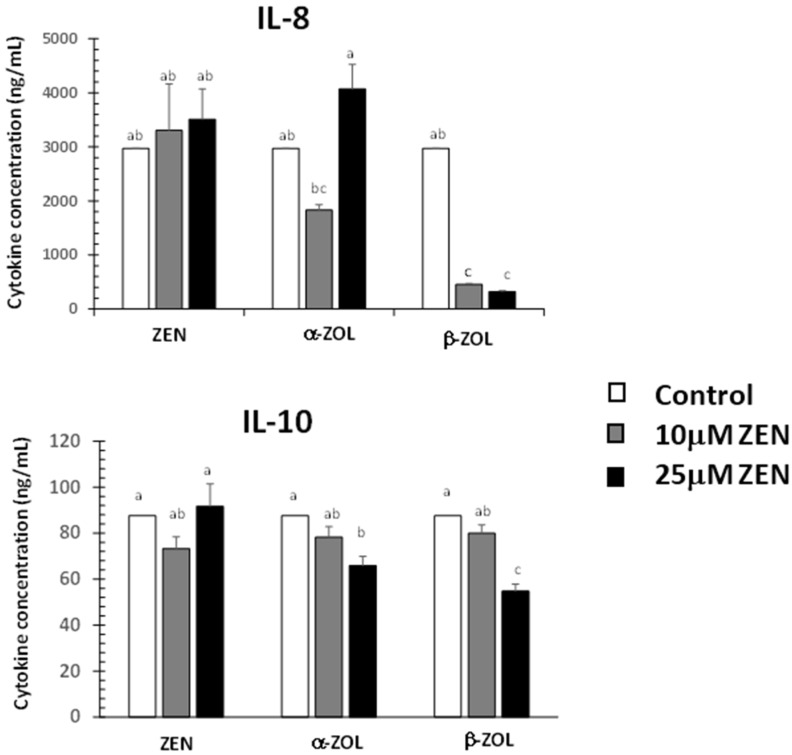
Effect of zearalenone and its metabolites on the cytokine synthesis in IPEC-1 supernatant*.* Cytokine synthesis was assessed in cell supernatant after 24 h of treatment with toxins at 37 °C. Data are means from four independent experiments. Two way ANOVA tests were performed to determine the effect of toxin treatment. Results are expressed as mean ± SEM for four different experiments, which are expressed as cytokine concentration (ng/mL). ^a–c^ indicate significant differences (*p* < 0.05) between control and ZEN and metabolites treated groups.

Intestinal permeability is regulated by different factors including exogenous factors, epithelial apoptosis, cytokines and immune cells [29]. For this reason we have analyzed the effects of ZEN and its metabolites on the synthesis of two cytokines: IL-8 and IL-10 (Figure 3).

Our results showed no effect of ZEN on IL-8 and IL-10 synthesis. Other studies showed that ZEN can increase the expression of toll like receptors and of certain cytokines involved in inflammation or responsible for the recruitment of immune cells [30]. By contrast, α- and β-ZOL decreased the expression of both IL-8 and IL-10 in a dose dependent manner, and, with the exception of IL-8, there was no effect produced by 10 µM of α-ZOL. Among ZEN metabolites, β-ZOL was the most toxic, inducing a decrease of the IL-8 synthesis by 84.8% at the dose of 10 µM and by 89.1% at the dose of 25 µM. In a similar way, β-ZOL induced a decrease of IL-10 synthesis by 8.8% and 37.5% at the dose of 10 µM and 25 µM, respectively. For 50 µM, the cytokine concentration was below the limit of detection (data not shown).

Intestinal immunity is assured by both immune and epithelial intestinal cells that cooperate in order to defend the body against pathogens, but also to avoid immune-mediated pathology in response to environmental changes and to maintain tissue homeostasis [1]. Epithelial cells can synthetize cytokines that have the potential to play an autocrine role or to influence adjacent non-epithelial cells [31]. IL-10 is a cytokine with multiple, pleiotropic, effects in immunoregulation and inflammation, with important anti-inflammatory effects [32].

As resulted from our data, ZEN metabolites decreased IL-10 synthesis as well as the intestinal permeability. Indeed, other results showed this similar evolution, because in mice, the treatment with IL-10 may prevent the increase in mucosal permeability during inflammatory disease [33].

Interleukin-8 (IL-8) is a common inflammatory factor that increases endothelial permeability during early stages of angiogenesis [34]. The increase of the intestinal barrier permeability may lead to intestinal inflammation [35]. In our study, IL-8 synthesis decreased similarly with the TER values after α-ZOL and β-ZOL treatment, but not after ZEN treatment. In a similar way, ZEN derivatives, but not ZEN, induced a significant decrease in IL-8 synthesis in swine polymorphonuclear cells [14]. Consequently, we can suppose that unlike ZEN, its metabolites have rather an anti-inflammatory effect on the epithelial intestinal cells.

In conclusion, our results showed that ZEN and its metabolites are able to differently affect porcine intestinal cell viability, transepithelial resistance and cytokine synthesis with important implication for the gut health.

## 3. Material and Methods

*Cell culture and reagents*: Intestinal porcine epithelial cell line (IPEC-1) derived from the small intestine of new born non-suckled piglets were kindly provided by Dr. P. Pinton, Laboratory of Toxicology-Pharmacology, INRA, France. IPEC-1 cells were grown and differentiated as previously described [19]. Purified ZEN, α-ZOL and β-ZOL (Sigma) were dissolved in ethanol/culture media (1:1, *v*:*v*), aliquated and stored at −20 °C before dilution in cell culture medium. The final concentration of the solvent was equal or less than 0.5%.

*Measurement of cell viability*: Cell viability in response to ZEN and ZEN metabolites was assessed through 2,3-Bis (2-methoxy-4-nitro-5-sulfophenyl)-2H-tetrazolium-5-carboxanilide inner salt (XTT) and neutral red (NR) assays by using Incytotox kit (Xenometrix, Switzerland). 2 × 10^5^ IPEC-1 cells were cultured in culture media, in 96 well plates, until reaching the confluence and then treated with different concentrations of toxins for 24 h. Cell viability was assessed according to the manufacturer’s instructions after the time of incubation. The stimulation index (SI) was expressed as the percent of the control cell. All tests were performed in four independent experiments. Doses that do not decrease cell viability under 90% (non-cytotoxic doses) were used for TER and cytokine assays.

*Measurement of transepithelial electrical resistance (TER) studies*: IPEC-1 cells were seeded at 10^5^ cells in culture media in 0.3 cm^2^ inserts with 0.4 mM pores (Becton Dickinson, Point de Claix, France). Cells reached confluence within 2 days. Differentiation media was then used and changed every other day until complete differentiation of cells (14 days). IPEC-1 cells were treated with 25 and 50 µM/L ZEN and ZEN metabolites and TER was measured for 10 days with a Millicell-ER Voltammeter (Millipore, Hessen, Germany). TER values were expressed as kW × cm^2^. The TER measurements were done in four independent experiments.

*Measurement of cytokine synthesis*: For cytokine assessment, IPEC-1 cells were seeded at 2 × 10^5^ cells in culture media in 24 wells plates, incubated for 24 h with concentrations of 10, 25, and 50 µM toxins; culture supernatants were collected and frozen at −20 °C until analyzed for cytokine content by a sandwich enzyme-linked immunosorbent assay (ELISA). Purified fractions of anti-swine cytokines (R&D Systems, Minneapolis, MN, USA): IL-8 (MAB5351), IL-10 (MAB6931), were used as capture antibody in conjunction with biotinylated anti-swine cytokines: IL-8 (BAF 535), IL-10 (BAF 693). Streptavidin-HRP (Biosource, Camarillo, CA, USA) and TMB (tetramethylbenzidine) were used for detection. Absorbance was read at 450 nm using a microplate reader (SUNRISE TECAN, Grödig, Austria). Recombinant porcine IL-8/CXCL8 (535-IN) and recombinant porcine IL-10 (693-PI) were used as standards and results were expressed as nanograms of cytokine per milliliter. All tests were performed in four independent replicates.

## 4. Statistical Analysis

A two way ANOVA test were used to analyze the differences in terms of cell viability, TER values and cytokine synthesis using the type of toxin and the toxin concentration as factors. The *p* values lower than 0.05 were considered significant.
